# Effects of Nodal Distance on Conditioned Stimulus Valences Across Time

**DOI:** 10.3389/fpsyg.2019.00742

**Published:** 2019-04-10

**Authors:** Micah Amd, Armando Machado, Marlon Alexandre de Oliveira, Denise Aparecida Passarelli, Julio C. De Rose

**Affiliations:** ^1^ Laboratory of Human Behavior Studies, Department of Psychology, National Institute of Science and Technology – INCT|ECCE, Federal University of São Carlos, São Carlos, Brazil; ^2^ Montreal Neurological Institute, McGill University, Montreal, QC, Canada; ^3^ Department of Psychology, University of South Pacific, Suva, Fiji; ^4^ School of Psychology, University of Minho, Minho, Portugal

**Keywords:** extinction, valence transformation, learning theory, classical conditioning, emotion

## Abstract

A meaningless symbol that repeatedly co-occurs with emotionally salient faces (US) can transform into a valenced symbol (CS). US-to-CS valence transformations have been observed for CS that have been directly (US→CS0) and indirectly (US→CS0→CS1→CS2) linked with face US. The structure of a US→CS0→CS1→CS2 series may be conceptualized in terms of “nodal distance,” where CS0, CS1, and CS2 are 0, 1, and 2 nodes from the US respectively. Increasing nodal distance between an evaluated CS and its linked US can reduce magnitude of observed CS valence transformations. We explored currently whether nodal distance can influence CS valence *extinction*, which describes reductions in CS valence following repeated exposures to CS without any accompanying US. In our study, faces with happy/neutral/sad expressions (US) were directly linked with nonsense words (US→CS0). The directly linked CS0 was concurrently linked with other words (CS0→CS1, CS1→CS2). Subjects evaluated all stimuli before and after conditioning, then continued to provide CS evaluations twice a week for 6 weeks. Bayesian factors provided credible evidence for the transformation and extinction of CS valences that were 0 and 1 nodes from US (all BF_10_’s > 100). The variability across post-conditioning CS evaluations provides indirect evidence for context-sensitive/propositional and structural/associative operations during CS evaluations.

## Introduction

Meaningless symbols can become emotionally salient following correlations with emotion-eliciting events (US) under controlled conditions (Staats and Staats, 1957; Mowrer, 1960; Osgood, 1980). For example, correlating nonsense words with pictures of happy faces (US) can transform the former into a positively valenced Conditioned Stimulus, or CS (Amd et al., 2018). CS valence transformations are not limited to CS that directly appeared with US only; in natural language, it is more often the case that words acquire valence following contextually mediated relations with other, valenced CS (Staats, 1961; Dymond and Rehfeldt, 2000; Tonneau, 2004; Amd et al., 2018). To see how, imagine the CS from the above example (call it CS0) was linked with a second term (call it CS1) in a context that promotes functionally equivalent stimulus relations/links (Tonneau, 2001). Establishing CS0-CS1 and CS0-US links can transform both CS0 and CS1 valences, despite CS1 having never directly co-occurring with US (Tonneau, 2004). In the latter case, the “indirect” relation between CS1 and US is mediated both by context and any intervening CS (Mowrer, 1960; Osgood, 1980; Fields and Verhave, 1987). The structure of US-CS relations can be characterized by “nodal distance” (Fields et al., 1990). Suppose a CS is paired directly with a US; call this relation US-CS0. Next, suppose our CS is paired with another nonsense word; call this CS0-CS1. To describe how “far” each CS is from its linked US, we say that CS0 and CS1 are 0 and 1 nodes from the US, respectively (e.g., Amd et al., 2018). Observing valence transformations for CS1 is an instance of indirect, 1-node transformation. The investigation of nodal distance effects may help us understand how derived representations relate to one other across psychological space to produce direct and indirect valence transformations (Fields and Verhave, 1987; Fields et al., 1990; Fields, 2016).

Indirect valence transformations have long fascinated psychologists interested in symbolic behavior since (most) linguistic symbols are thought to become salient through similar transformations (Mowrer, 1954; Osgood and Tzeng, 1990; Dickins and Dickins, 2001; more recently, see investigations in “relational frame theory”—Dymond and Roche, 2013; Amd and Roche, 2015). We know that when CS and US categories consist of nonsense words and emotional faces respectively, valence transformations appear limited by nodal distance. Specifically, valences for CS that are two or more nodes away from their face US may not significantly transform (Bortoloti and de Rose, 2009; Amd et al., 2018; Silveira et al., 2018). A recent study by Amd et al. (2018) illustrates this point.

**Figure 1:**
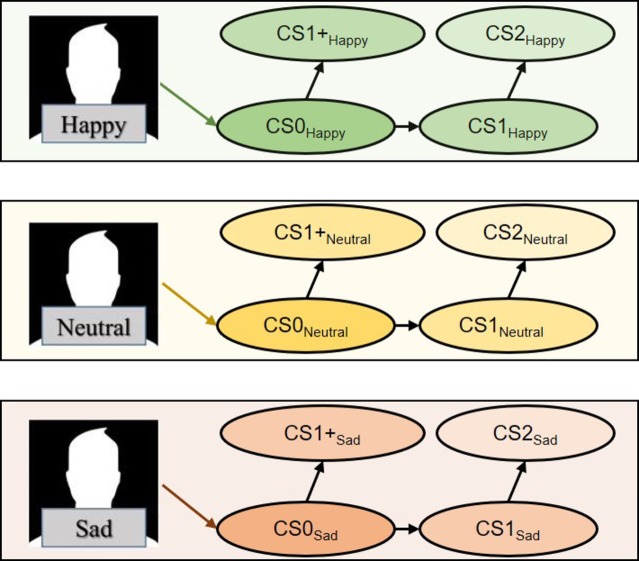
Subjects were presented with US-CS and CS-CS sequences designed to establish stimulus sets with CS that were 0, 1, and 2 nodes apart from their respective USs. Stimuli linked together during training are connected by arrows. For instance, faces were exclusively linked with CS0. CS0 was linked equally with CS1 and CS1+. Finally, CS1 was linked with CS2.

**Figure 2:**
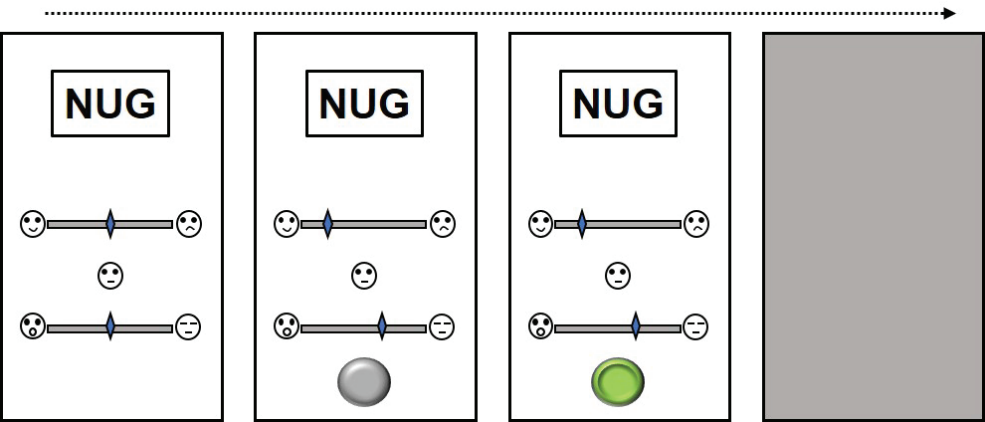
Subjects evaluated CS and distractors (represented by the meaningless word “NUG”) using valence and arousal sliders scored between 0 (sad/calm) and 100 (happy/aroused). Each trial commenced with both sliders set at 50 (Neutral). Subjects were required to move both sliders before a gray button would appear near the bottom of the screen. Pressing the button turned it green, produced a jittered blank interval followed by the next evaluation trial.

In that study, subjects were trained along US→CS0, CS0→CS1, and CS1→CS2 relations, producing CS that were 0, 1, and 2 nodes away from emotional face US respectively (Figure 1). All stimuli were evaluated before and after conditioning on visual-analogue scales (VASs) corresponding to emotional dimensions of valence and arousal (Figure 2). Those authors reported significant valence transformations for CS0 and CS1 and marginal effects for CS2, which was 2 nodes from the US (p. 12). Those findings raised the question as to whether nodal distance may influence the *extinction* of CS valences (Baeyens et al., 1988; Baeyens et al., 1995). Briefly, “extinction” describes how repeated presentations of a CS alone in a context where it had previously been associated with a US may cause the CS valences to trend toward their pre-conditioning state. We replicated Amd et al. (2018) procedure for establishing three 5-member stimulus sets that varied along valence (positive/happy, neutral, negative/sad). We expand on Amd’s work by having subjects evaluate CS intermittently for approximately 2 months to determine how CS were evaluated across time. Reductions in CS valence would indicate extinction.

Before describing our study further, it is worth noting that in human evaluative learning, the prediction that repeated exposures to nonreinforced CS will cause their valences to extinguish has not always been met (Hofmann et al., 2010). In a representative study by Hermans et al. (2002), pictures of human faces (CS) were linked with aversive electric shock (US) for human subjects. After face→shock (CS→US) acquisition trials, some subjects immediately evaluated CS. Other subjects first underwent a block of extinction trials, where CS appeared without accompanying shocks, and were then asked to evaluate CS. Despite the latter group undergoing extinction, Hermans reported no significant group differences between CS evaluations. The retention of CS valence following CS-alone trials was interpreted to mean that CS valences were “resistant” to extinction. Those authors also collected US expectancy ratings, where subjects’ responding was found to cohere with learning histories. Specifically, US expectancy was high following CS→US (acquisition) trials, and low following CS-alone (extinction) trials. This disassociation between US expectancy (high/low) and CS valence (high/high) has been proposed as evidence for the dual operation of propositional and associative processes during CS valence transformations (Bar-Anan and Moran, 2017; Gawronski and Bodenhausen, 2018; McLaren et al., 2018).

According to a dual-process view, US expectancy ratings correlate with the relational information provided by the contextualized (propositional) relationship between the CS and US (e.g., CS *predicts* US, CS *does not predict* US, CS *is different from* US). On the other hand, without a context constraining evaluation strategies, collected CS valences may (or not) resemble their linked US, independent of relational information available (Jones and McLaren, 1999; Sweldens et al., 2014). Some supporting evidence comes from Moran and Bar-Anan (2013), who conditioned images of cartoons (CS) with positively/negatively valenced sounds/words (US) for their subjects. A key manipulation was the sequence of CS-US presentations, where some CS anticipated US onset (CS→US) while other CS correlated with US offset (US→CS). Following conditioning, subjects evaluated CS using explicit and implicit evaluation measures. Across explicit evaluations, subject performances corresponded with the relational information provided—for example, CS which signaled the offset of negative US were explicitly preferred over CS which signaled the offset of positive US. When looking across implicit evaluations however, CS linked with negative US were always evaluated as negative, regardless of whether they had signaled US onset/offset during conditioning. The latter implies the operation of an associative process by which the “mere” correlation of a CS with a valenced US would suffice *ipso facto* to transform CS valences, particularly if the capacity to engage in propositional deliberation is dampened (Bar-Anan and Moran, 2017). The disassociation between explicit and implicit CS evaluations provides further evidence of dual processes underlying CS valence evaluations (McLaren et al., 2014, 2018; Sweldens et al., 2014).

The hypothesis that dual processes contribute toward the CS valence transformation and extinction was recently challenged by Aust et al. (2018), who suspected that the expectancy-valence disassociation may be an artifact of contextually cued judgment strategies (rules). Aust et al. reasoned that acquisition (CS-US) and extinction (CS-alone) contexts cue different rules for evaluating CS valence and US expectancy based on the learning histories associated with the respective contexts. Those authors tested this hypothesis by having subjects undergo acquisition and extinction in separate contexts. After conditioning, different groups provided CS valence and US expectancy ratings in acquisition or extinction contexts. Evaluations were also collected across contexts that combined features of both acquisition and extinction (called an “integrated” context), as well as novel contexts. Subjects who evaluated CS valence and US expectancy in the acquisition context (associated with CS→US) produced high valence and expectancy values. Alternatively, subjects who evaluated CS in the extinction context (associated with CS-alone) produced low valence and expectancies, supporting those authors’ predictions. Alternatively, if US expectancies were collected in an acquisition context and CS valence in the alternate extinction context (or vice versa), the familiar expectancy-valence disassociation re-appeared (Aust et al., 2018, p. 27). Specifically, valence was high (resistant to extinction) when subjects evaluated CS in acquisition contexts; valence was low (extinction) when CS were evaluated in extinction contexts. CS valences in novel and integrated contexts fell in between. Aust reasoned that the latter ratings were consequated by an integration of learning histories associated with acquisition and extinction contexts. While this could explain CS evaluations in the integrated context, the generalizability of this explanation to CS valences collected in novel contexts is less convincing. In any case, the results reported by Aust et al. (2018) suggest that expectancy-valence disassociations in earlier studies may have been driven by different, context-sensitive judgment strategies for evaluating CS valences and US expectancies. We considered Aust’s findings during the design of the present study (described below), where all CS evaluations were collected in a context that did not resemble the learning context.

In our study, subjects underwent a relational learning (conditioning) task for establishing three 5-member series structured along US→CS0, CS0→CS1, CS0→CS1+, and CS1→CS2, where US consisted of happy/neutral/sad faces (Figure 1). Before and immediately after conditioning, subjects evaluated stimuli along valence and arousal VASs (Figure 2). Subjects continued evaluating CS twice a week for 6 weeks. Earlier proof-of-concept studies revealed that varying the interval between CS evaluations reduced the likelihood of control by temporal cues, as subjects could not predict which days they would be probed for CS evaluations (e.g., Matute et al., 2011). After the final evaluation, subjects returned to the lab to complete a set of card-sorting tasks that were unrelated to the present investigation. Three hypotheses were currently investigated. First, we predicted that the valences of CS both directly and indirectly linked to USs would transform following conditioning. Second, the magnitude of the CS valences will inversely relate to increasing nodal distance from US (Amd et al., 2018). Finally, post-conditioning CS valences trending toward their pre-conditioning values would suggest extinction.

## Materials and Methods

### Subjects

Fourteen college students (12 females, 18–24 years) participated in the present study. Subjects were informed they would be participating in a study on “emotional memory” and provided no additional details until the end of their participation. All subjects provided written and informed consent prior to participating. Our study corresponds with the principles described in the Declaration of Helsinki. All procedures reported were approved by the ethics committee on human research at the Federal University of Sao Carlos. All subjects received R$50.00 (USD15.50) for participation. None of the subjects were familiar with the task before the procedure.

### Materials

Fourteen trigrams were randomly assigned to be CS (12) or distractors (2) for each subject. Specifically, our program randomly selected 12 items from an array of 14 trigrams provided as input for each subject. No two subjects viewed the exact same 12 trigrams as CS. Distractors appeared only during the stimulus evaluation task. Nine emotional faces (three happy, three neutral, three sad) from the Karolinska face database (Goeleven et al., 2008) were used as US. During pre-training (see Procedure), we used nine images of real objects (apple, banana, melon, car, bus, motorbike, hammer, wrench, pliers). All materials were the same as those reported in Amd et al. (2018). All evaluations were collected on a touch screen application developed on LiveCode (v. 6.7) platform and installed on subjects’ smartphones for the duration of the study (Figure 2). The conditioning task was designed and implemented on E-Prime 2 (Schneider et al., 2012) on a Windows laptop with a 43-cm high-definition screen. All data analyses were conducted on the freely available JASP software (JASP, 2018). All subjects underwent conditioning in quiet temperature-controlled rooms in the Laboratory of Human Behavior Studies (in Portuguese—LECH) at the Federal University of Sao Carlos. The locations for subsequent stimulus evaluations were contingent on the subjects’ whereabouts during the time of ratings.

### Procedure

#### Baseline Evaluation

After receiving verbal and written consent, the experimenters installed the evaluation task on subjects’ smartphones. The task commenced with an instruction screen and a button labeled START. Pressing this would begin the trial sequence illustrated in Figure 2. On each trial, a CS/distractor would appear in the screen center with two VASs underneath. Subjects had to move each slider, one anchored by sad and happy faces (representing valence) and the other by calm and excited faces (representing arousal). Slider positions were scored from 1 (sad/calm) to 100 (happy/excited). Moving both sliders produced a gray button near the bottom of the screen. Pressing this produced the next trial. A blank screen, jittered between 0.5–1.5 seconds, seperated evaluation trials. We jittered our interval to prevent temporal conditioning artifacts (Balsam, 1984). CS presentation sequences were randomized for each iteration of the evaluation task. A black screen with a button labeled FINISH would appear after all evaluations were complete. Pressing this emailed the stimulus evaluations to a secure server and terminated the application. After collection of these baseline evaluations, the subject commenced the conditioning phase of the experiment on a laptop.

#### Conditioning

Subjects underwent 60 pre-training trials followed by 180 conditioning trials using the procedure described in Amd et al. (2018). During pre-training, subjects viewed naturally congruent stimulus sequences (e.g., melon→banana, apple→banana) followed by a card-sorting test where they had to pair congruent stimuli together (e.g., “apple” with “melon”). The pre-training phase was designed to help subjects understand task demands (Amd and Roche, 2017). During conditioning, subjects viewed face→word (US→CS0) and word→word (CS0→CS1, CS0→CS1+, CS1→CS2) sequences across 180 trials in randomized order. Three faces from each US category (happy/neutral/sad) were correlated with a single CS0 5 times across 15 trials. We used multiple US since single exemplar US-to-CS mappings are insufficient for transforming CS valences (Gawronski et al., 2017, Experiment 1; also see Dube et al., 1992). CS0 was linked with CS1 over 15 trials, CS0 with CS1+ over 15 trials, and CS1 with CS2 over 15 trials. CS1 and CS1+ were both 1 node away from the US, where CS1+ functioned as an end-term. This allowed us to check whether end-terms (CS1+) are differentially evaluated relative to intermediate terms (CS1) during extinction, since no differences are expected during acquisition (Amd et al., 2018). After conditioning, all subjects produced card pairs from a deck containing all CS0 and CS2 stimuli as a test for contingency awareness for an unrelated investigation. As contingency awareness may not be critical for CS valence transformations (Amd and Baillet, 2019) and the present investigation was not concerned with sorting/transitivity performances anyway (but see Amd and Roche, 2017; Amd et al., 2018), no further mention of sorting performances are provided.

#### Post-evaluations

All subjects immediately evaluated stimuli after conditioning. Afterward, subjects would receive a message stating “*um lembrete* (a reminder)” between 3 PM and 4 PM twice a week for the next 2 months. The message functioned to cue completion of the evaluation task installed on subjects’ smartphones. All post-conditioning CS evaluations were collected within 1–3 h of sending the prompt. We varied the day on which CS evaluations were collected to reduce temporal discriminative control (Savastano and Miller, 1998). The collection of 13 additional evaluations marked the end of the current experiment.

#### Data Analyses

For all subjects, CS evaluations were collected over 14 time points (t1*t2*t3…t14), where baseline/pre-conditioning evaluations were collected at t1. CS were parsed by nodal distance (CS0*CS1*CS1 + *CS2). CS evaluations collected between time points t1 and t2 were contrasted with Bayesian paired *t* tests, where significant effects would inform whether CS valences transformed. We next calculated difference scores between t1 and t2 for all CS and subjected the data to three analyses of variance (ANOVAs), one per valence condition (happy, neutral, sad) with nodal distance (4) as the independent factor. Significant effects would highlight whether nodal distance modulated valence transformation for that condition. To measure extinction, we ran three 4*13 mixed ANOVAs for each valence condition (happy, neutral, sad) with nodal distance (4) and post-conditioning time (13) as independent and repeated factors respectively. Significant interactions were followed with tests for simple main effects and Bonferroni-corrected *post hoc* contrasts. Prospective power analyses with GPower 3 (Faul et al., 2007) recommended sample sizes for a large effect (*f* = 0.5), with alpha and power set at *p* = 0.05 and 1–β = 95% respectively. Assuming nonsphericity across all time points (epsilon = 1), the recommended sample for a 4*13 ANOVA was 12 participants and a critical F (*F_Crit_*) of 1.54. Assuming sphericity was violated at all time points, the nonsphericity correction would be (1/(1–13)) = − 0.08 (p. 181). In that case, the recommended sample size with all remaining parameters would be 32, *F_Crit_* = 2.73. As we did not know our sampling distribution *a priori*, we chose an intermediate epsilon of 0.5, which then yielded a recommended sample size of 12 subjects with *F_Crit_* = 1.82.We complemented our ANOVAs with Bayes factors (BFs) estimated to a precision of ±2% and computed using a standard Cauchy prior of width 0.707 and a scaling parameter of 0.5 for fixed effects (Rouder et al., 2012). BFs evaluate the quality of evidence underlying an observed effect relative to the non-effect predicted by the null hypothesis for that same data set (Kruschke, 2013). We used BF_10_ here to qualify the evidence for any statistically significant effects (e.g., 3 > BF_10_ > 1 = weak; 10 > BF_10_ > 3 = moderate; 30 > BF_10_ > 10 = strong; BF_10_ > 30 = very strong). All reported *F* values were Greenhouse–Geisser corrected when appropriate.

## Results

### US-to-CS Valence Transformations

**Figure 3:**
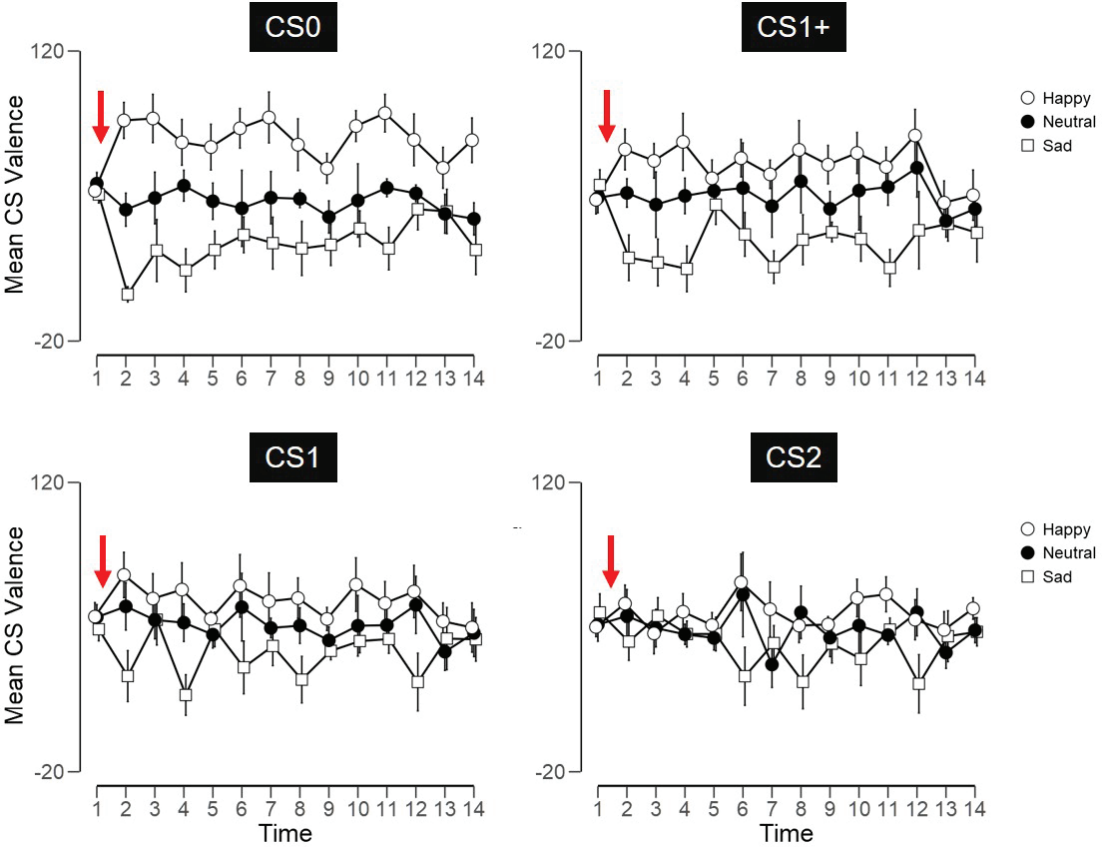
CS valences were collected across 14 time points (*x*-axis) and were scored from 1/sad to 100/happy (*y*-axis). Conditioning took place between times 1 and 2 (red arrow). All subsequent evaluations were spaced 2–3 days apart. Error bars indicate 95% confidence intervals.

We found no convincing evidence for significant transformation effects across any of the CS arousal ratings (all *p’*s > 0.05; all BF_10_’s < 2). Subsequent analyses are reported for CS valences only (Figure 3). Contrasting evaluations between pre- and post-conditioning time points (time 1 vs. 2—Figure 3) revealed significant effects for CS0_Happy_ (*t* = 8.2, *p* < 0.001, *d* = 2.18, BF_10_ > 1,000) and CS0_Sad_ (*t* = 20.9, *p* < 0.001, *d* = 5.59, BF_10_ > 1,000), i.e., for CS directly linked with happy and sad faces. Contrasts for CS associated with neutral faces were not significant (*p* > 0.05). For CS indirectly linked to US, significant effects appeared for CS1_Happy_ (*t* = 5.2, *p* < 0.001, *d* = 1.39, BF_10_ = 177); for CS1+_Happy_ (*t* = 3.9, *p* = 0.002, *d* = 1.03, BF_10_ = 22); for CS1_Sad_ (*t* = 7.3, *p* < 0.001, *d* = 1.96, BF_10_ > 1,000); and for CS1+_Sad_ (t = 7.5, *p* = 0.002, *d* = 1.06, BF_10_ = 26). The differences for CS2_Happy_ (*p* = 0.015, *d* = 0.75) and CS2_Sad_ (*p* = 0.037, *d* = 0.62) were statistically significant, but not credible (all BF_10_’s ≤ 4.1). Significant ANOVAs across CS in the happy (*F* = 4.68, *p* = 0.006, $\eta_{p}^{2}$ = 0.21, BF_10_ = 8) and sad (*F* = 9.09, *p* < 0.001, $\eta_{p}^{2}$ = 0.34, BF_10_ = 396) conditions illustrate an inverse relation between increasing nodal distance and CS valence magnitude.

### Effects of Nodal Distance on Conditioned Stimulus Extinction

4*13 ANOVAs for each valence condition (happy, neutral, sad) produced significant effects of time appeared for all conditions (all *p*’s < 0.001, all BF_10_’s > 1,000). Nodal distances interacted significantly with time for happy (*F* = 1.59, *p* = 0.017, $\eta_{p}^{2}$ = 0.08, BF_10_ > 1,000) and sad (*F* = 6.45, *p* < 0.001, $\eta_{p}^{2}$ = 0.11, BF_10_ > 1,000), but not neutral (*p* = 0.672) conditions. Correspondingly, significant nodal distance effects appeared for CS linked with happy (*F* = 23.7, *p* < 0.001, $\eta_{p}^{2}$ = 0.58) and sad (*F* = 13.8, *p* < 0.001, $\eta_{p}^{2}$ = 0.44), but not neutral (*p* = 0.118) faces. *Post hoc* tests across negative/sad CS revealed significant (*p* < 0.005) differences between CS0 and CS1, and between CS1 and CS1+. Across positive/happy CS, *post hoc* tests revealed CS0 to be significantly (*p* < 0.001) greater than all remaining CS (CS1, CS1+, CS2). No other contrast reached significance (all *p*’s > 0.05).

## Discussion

Subjects underwent a conditioning task where nonsense words (CS) were concurrently linked with happy/neutral/sad faces (US→CS0) and other nonsense words (CS0→CS1, CS0→CS1+, CS1→CS2). Subjects evaluated CS before and after conditioning, then provided 13 additional CS evaluations over 6 weeks. Comparison of CS valences before and after conditioning confirmed earlier reports by Amd et al. (2018), who reported significant valence transformations for CS that were 0 and 1 nodes removed from happy and sad US. Analysis of the variances across post-conditioning evaluations provided evidence for positive and negative CS valences trending toward pre-conditioning values, moderated by nodal distance.

Our investigation was not designed to compare dual-process and single-process accounts, but they raise some questions regarding the account provided by Aust et al. (2018). According to those authors, CS extinction measurements reflect the “integrative summaries of the learning history” (p. 28) which, during the present study, constituted of acquisition trials only. Our findings partially support Aust’s claim, in that the first collection of CS evaluations immediately after conditioning suggested that subjects were integrating valence information following acquisition (time 2, Figure 3). The issue arises when trying to account for the significant variability across remaining evaluations, particularly for CS indirectly linked to US (time 3 onward, Figure 3). If evaluations are consequated by judgment strategies/rules bound to specific contexts, how would evaluating the same CS in the same context evoke variable ratings? Alternatively, if subjects attribute qualifiers to familiar CS and these determine CS evaluations (as per De Houwer, 2018), variability across CS valence should have stabilized across time, whereas our observations indicate the exact opposite. On balance, an unpublished pilot study preceding the reported work showed subjects who evaluated CS daily (instead of every 2–3 days, as was the case here) repeated the values provided during the first post-conditioning evaluation across all remaining evaluations. Those subjects were shown to accurately recall CS-US contingencies from acquisition after all post-conditioning evaluations were complete. That study indicated CS evaluations can reflect integrative summaries of earlier learning histories, *pro* Aust et al., albeit with the caveat that acquisition and extinction learning histories can be subjectively delineated post-conditioning.

In contrast, subjects in the current study could not recollect which CS and US “went together” during an informal post-experiment interview. While there is some evidence that CS valences may transform without explicit knowledge of CS-US contingencies (e.g., Hütter et al., 2012), we cannot answer whether this was presently the case since we do not know if/when CS-US contingency knowledge was “forgotten” post-conditioning. Relatedly, although our evaluation context differed from the learning context, we could not control the context where subjects provided the ratings, nor their subjective state at the moment of the ratings. The confluence of contextual and subjective factors may have contributed to the variability observed across post-conditioning evaluations. A future work could address both issues by (1) interspersing CS evaluations with tests of contingency awareness and (2) collecting post-conditioning evaluations in standardized contexts. The first manipulation will determine whether retention of acquisition contingencies influences post-conditioning variability in CS valence (e.g., extinction). The second manipulation would mitigate the influence of interpersonal contextual differences.

An additional point involves nodal distance effects reported here and in earlier works (e.g., Bortoloti and De Rose, 2009; Amd et al., 2018). A central feature of those studies was the demonstration of untrained transitive relations. For example, imagine we train a competent subject along the propositions “*A* goes with *B”* and “*B* goes with *C*.” If our hypothetical subject can derive “*A* goes with *C*” or “*C* goes with *A*” without additional training, one could assume *A*, *B*, and *C* are functionally equivalent (Tonneau, 2001). Now imagine that *A* consists of happy faces while *B* and *C* are meaningless words, as was the case presently. All else remaining equal, why would *B* and *C* evoke non-equivalent valences? These effects can be accommodated by validation processes operating alongside spreading activations across contextually specified mediators (e.g., Bar-Anan and Moran, 2017; Gawronski and Bodenhausen, 2018; then see Berlyne, 1965). Is the explanation from a single-process perspective “better” (in terms of parsimony and falsifiability)? Deriving explanations through assumptions incorporating propositional processes exclusively can be parsimonious, but with the considerable cost that single-process models are “virtually immune to falsification” (De Houwer, 2018, p. 14). It may be more fruitful to hypothesize structural/associative and context-sensitive, propositional processes interact to produce CS evaluations (Berlyne, 1965). We already have evidence of bottom-up valence effects during time windows too early to attribute to propositional reasoning processes (Amd et al., 2013; Bayer et al., 2017; Amd and Baillet, 2019). To be fair, advocating for dual processes requires a satisfactory explanation of how propositions arise from, as well as interact with, structures of associative S-S links, for which we have no satisfactory response presently (De Houwer, 2018; although see Gawronski and Bodenhausen, 2018). Yet, associative models can already account for the emergence of transitive relations following direct training of linear stimulus relations without requiring formal representations of relational qualifiers (Tovar and Westermann, 2017). A valuable next step could be to determine whether CS valence transformations can be modeled without formal representations of relational qualifiers.

We conclude by noting some limitations of the present work. First, some may criticize our exclusion of implicit CS evaluation and US expectancy measures, both of which are common across investigations of CS valence extinction (e.g., Hu et al., 2017). We had no reason to include US expectancy measures as task demands across evaluation and acquisition contexts were established *a priori* as different. No CS-US sequences appeared during post-conditioning evaluations, hence there was no reason to expect US in the first place. We excluded implicit CS evaluation measures for three reasons. First, we were concerned with poor reliability when repeated assessments are made with implicit tests (Buchner and Wippich, 2000). Second, merely identifying a CS (such as during some implicit evaluation task) can cause subjects to derive CS-valence links unrelated to explicitly provided CS-US information (cf., the misattribution effect—Jones et al., 2009). This means that *any* (explicit/implicit) measure of CS valence could potentiate the derivation of CS valences unrelated to task demands (Jones et al., 2009; Hahn and Gawronski, 2018). For fairness, the same argument applies to CS evaluations reported here since we did not assess whether subjects misattributed valences to CS. A future replication could nevertheless incorporate implicit evaluation measures to determine whether repeated evaluations potentiate CS valence misattributions that manifest across explicit evaluations. A second criticism can be raised regarding the limited power afforded by our sample size (*n* = 14). While other research should replicate our procedure with larger samples, the incorporation of Bayesian factors alongside our frequentist analyses implies our depicted effects as highly credible. It is worth asking whether the present effects would be replicated with symbolic CS incorporating US across various modalities (e.g., visual/olfactory/auditory). Some important work in this area has been conducted by relational frame theorists, who have demonstrated the transformation of CS response properties beyond valence (e.g., Dymond and Rehfeldt, 2000; Dymond and Roche, 2013). Observing extinction, or a lack thereof, of other response properties would highlight the extent to which the present effects may be universal to symbolic conditioning investigations, and relational categorization in general (cf., Goldwater and Schalk, 2016). It could also be the case that our reported effects are limited to preparations involving emotional faces and meaningless words. Answering these questions will clarify the confluence of structural and propositional processes during CS transformation and extinction.

## Ethics Statement

This study was carried out in accordance with the recommendations of the university ethical review committee for human research. All subjects gave written informed consent in accordance with the Declaration of Helsinki. The protocol was approved by the Federal University of Sao Carlos ethical review committee for research on human subjects.

## Author Contributions

MA designed the study, prepared the manuscript, and analyzed the data. MAO and DAP performed the data collection. AM and JR assisted with manuscript preparation.

### Conflict of Interest Statement

The authors declare that the research was conducted in the absence of any commercial or financial relationships that could be construed as a potential conflict of interest.
