# Corrigendum: Effects of Nodal Distance on Conditioned Stimulus Valences Across Time

**DOI:** 10.3389/fpsyg.2019.01253

**Published:** 2019-05-28

**Authors:** Micah Amd, Armando Machado, Marlon Alexandre de Oliveira, Denise Aparecida Passarelli, Julio C. De Rose

**Affiliations:** ^1^Laboratory of Human Behavior Studies, Department of Psychology, National Institute of Science and Technology – INCT|ECCE, Federal University of São Carlos, São Carlos, Brazil; ^2^Montreal Neurological Institute, McGill University, Montreal, QC, Canada; ^3^Department of Psychology, University of South Pacific, Suva, Fiji; ^4^School of Psychology, University of Minho, Minho, Portugal

**Keywords:** extinction, valence transformation, learning theory, classical conditioning, emotion

In the published article, there was an error regarding the affiliation for “Julio C. De Rose.” Instead of affiliation “3” it should have been affiliation “1.”

The authors apologize for this error and state that this does not change the scientific conclusions of the article in any way. The original article has been updated.

